# Investigating the mechanism of PAD in the treatment of acne based on network pharmacology and molecular docking: A review

**DOI:** 10.1097/MD.0000000000038785

**Published:** 2024-07-19

**Authors:** QianJun Yan, Fang Zhang, Zukang Qiao, Yangzi Jin, Ruyi Zheng, Jiani Wu

**Affiliations:** a The First Affiliated Hospital of Zhejiang Chinese Medical University (Zhejiang Provincial Hospital of Chinese Medicine), Hangzhou, China; b Department of Integrated of Traditional Chinese and Western Medicine, Anqing 116 Hospital, Anqing, Anhui, China; c Department of Otorhinolaryngology, The First Affiliated Hospital of Zhejiang Chinese Medical University (Zhejiang Provincial Hospital of Chinese Medicine), Hangzhou, Zhejiang, China.; d Department of Otorhinolaryngology, Hangzhou TCM Hospital Affiliated to Zhejiang Chinese Medical University, Hangzhou, Zhejiang, China

**Keywords:** acne, molecular docking, network pharmacology, powder for ascending and descending

## Abstract

Acne is a common and chronic skin condition characterized by high incidence, recurrent symptoms and difficult cure. Summarizing the clinical treatment experience, it was found that the powder for ascending and descending was effective in the treatment of acne. Our aim was to use network pharmacology and molecular docking to reveal the hub genes, biological functions, and signaling pathways of powder for ascending and descending against acne. First, the chemical components and target genes of PAD were sifted using the TCMSP and HERB database. The targets of acne were obtained simultaneously from the CTD, OMIM and GeneCards database. The obtained drug targets and disease targets were imported into the R language software to draw Venn diagrams. Then, the potential targets were imported into the String website to construct a protein interaction network diagram. And Cytoscape software was used for topological analysis to screen the core targets, and the core targets were analyzed by GO functional enrichment and KEGG pathway enrichment. Finally, molecular docking was used to verify the predictions of key genes’ reliability. The core targets of the treatment of acne were TNF, GADPH, IL-6 and so on. The results of enrichment analysis showed that the treatment of acne with PAD may be related to TNF signaling pathway and AGE-RAGE signaling pathway. The molecular docking verification showed that the components were well bound to the core targets of acne, and the docking ability of stigmasterol and TNF (−12.73 kcal/mol) was particularly outstanding.

## 1. Introduction

Acne is a chronic inflammatory skin disease that occurs in the sebaceous glands of the hair follicles, and its etiology is complex and is related to the level of male hormones in the body, the secretion of the sebaceous glands, the enhancement of the keratinization process in the infundibular part of the hair follicle, and the bacterial proliferation of Propionibacterium acnes in the hair follicles of the face, neck, chest, and back.^[1]^

The incidence of acne in adolescents is 97.8% of boys and 89.8% of girls aged 16 to 18 years, and the prevalence of men is higher than that of women, usually starting before puberty and continuing into adulthood, and some patients can also extend to more than 30 years old.^[1]^ According to data from the 2013 Global Burden of Disease Study, acne accounts for 0.29% of all skin diseases and 1.79% of the global burden of disease, and acne ranks second among the most common skin diseases, after dermatitis.^[2]^ The economic burden of acne in the United States is estimated at $300 million per year, and the pathogenesis of acne is complex, and it is prone to recurrence, making it difficult to achieve the desired treatment goals. Acne can affect the aesthetics of a patient’s face and have a profound negative impact on a patient’s mental health, including social isolation, decreased self-esteem, and increased anger, and are significantly associated with suicidal ideation.^[3]^

The main treatments for acne include skin care (including frequency of washing, type of cleanser, moisturizer), topical medications (including retinoids, antibiotics, benzoyl peroxide, salicylic acid), oral antibiotics (such as doxycycline or minocycline), and hormones (such as combined oral contraceptives or spironolactone).^[4]^ However, the above treatments are generally ineffective and have certain adverse effects.

Traditional Chinese medicine (TCM) believes that damp-heat toxin are the main cause of acne.^[5]^ TCM is able to improve the symptoms of acne by removing heat toxins and moisture.^[6]^ As a second-line treatment drug, traditional Chinese medicine has played an important role in the treatment of acne, with the advantages of a long history, low toxicity and small side effects.^[7]^

Powder ascending and descending (PAD) originated from the Qing Dynasty physician Yang Lishan’s “Typhoid Plague Strips,” its ingredients include 6 g of white stiff silkworm, 3 g of cicada slough, 9 g of turmeric, and 12 g of rheum officinale (raw), the main effect of PAD is to relieve the stagnation of qi and dissipate toxic heat, and reconcile qi and blood. Because the PAD has the dual effect of “upward and outward publicity” and “downward and inward discharge,” it is called PAD. Modern Chinese medicine treats acne, and most of the syndromes are characterized by heat exuberance in lung and stomach. According to the theory of “dissipate excessive stagnation of fire”, PAD has the effect of dissipating heat and is very effective in acne treatment.

By summarizing the clinical treatment experience, it was found that PAD had good efficacy and few adverse reactions in the treatment of acne, and this article aimed to explore the potential mechanism of action of PAD in the treatment of acne by using network pharmacology and molecular docking technology, so as to provide theoretical support for the clinical treatment of acne.

## 2. Materials and methods

### 2.1. Screening of active ingredients of PAD and prediction of potential targets

All the chemical components and targets of rheum officinale, turmeric, white stiff silkworm, and cicada slough were searched through Traditional Chinese Medicine Systems Pharmacology (TCMSP, https://old.tcmsp-e.com/tcmsp.php) and HERB (http://herb.ac.cn/) databases. According to the pharmacokinetic parameters, the oral bioavailability of the drug was set to ≥ 30% and the drug-like index (DL) ≥ 0.18 as the screening conditions, and the corresponding target proteins of each active ingredient were obtained. The Uniprot database (https://www.uniprot.org/) was used to normalize the above target proteins, and the non-human target was excluded.

### 2.2. Targets acquisition for acne

With “acne” as the keyword, the three databases of the GeneCards human gene database, Comparative Toxicogenomics database, and Online Mendelian Inheritance In Man database were searched to obtain the relevant disease target genes that entered into these databases before December 1, 2023, and then the targets obtained from the three databases were sorted out and the duplicate targets were deleted.

### 2.3. Network construction, analysis and comparison of PAD in the treatment of acne

By analyzing the above-mentioned drug targets and disease targets and intersecting them, the potential targets of PAD for the treatment of acne were obtained, and the Venny 2.1 online tool was used to draw a Venny diagram. The potential targets were imported into the String database, the protein type was set as homo sapiens, the highest confidence protein parameter score was ≥ 0.4, the free protein was hidden, and the protein interaction relationship in TSV format was obtained, and then the protein-protein interaction (PPI) network diagram was constructed into Cytoscape3.8.0 software, and the core targets of PAD for the treatment of acne were further screened, and the “drug-component-core target-disease” network was constructed.

### 2.4. Functional enrichment analyses

The gene ontology (GO) and Kyoto encyclopedia of genes and genomes (KEGG) enrichment analysis of the core targets were carried out by R language software, and the possible biological functions and main signaling pathways of PAD in the treatment of acne were explored, and the *P* value < .05 was set as the standard, and the enrichment results with significant differences were screened according to the *P* value, and the GO biological functions and the top 20 KEGG signaling pathways with enrichment results were output respectively.

### 2.5. Verification of molecule docking

Download the 2D structure of the key active ingredients of PAD from the PubChem database (https://pubchem.ncbi.nlm.nih.gov) and save it in SDF format. Download the 3D structure of the core target proteins from the Protein Data Bank database (https://www.rcsb.org/) and save it in Protein Data Bank format. The water molecules and small molecule ligands were removed by Pymol software, and then hydrogenated by AutoDockTools software, and the acceptor and ligand were molecularly docked, and their binding activity was evaluated by the docking energy value.

## 3. Results

### 3.1. Screening of active ingredients and targets prediction of PAD

Under the screening conditions of oral bioavailability ≥ 30% and DL ≥ 0.18, a total of 74 active ingredients of PAD were screened by TCMSP and HERB databases, including 22 active ingredients of rheum officinale, 3 active ingredients of turmeric, 18 active ingredients of cicada slough and 31 active ingredients of white stiff silkworm. A total of 1159 targets were predicted by the Uniprot database, including 127 rheum officinale, 34 turmeric, 416 cicada slough, 582 white stiff silkworm, and 793 predicted targets were obtained after sorting. Due to the large number of active ingredients in each herb, only some of the active compounds and gene targets are listed (Table 1).

**Table 1:** Drug-active compounds and gene targets.

TCM	MOL ID	Molecule name	Gene
Rheum officinale	MOL000471	aloe-emodin	PTGS1
Rheum officinale	MOL000471	aloe-emodin	PTGS2
Rheum officinale	MOL000471	aloe-emodin	HSP90AB1
Turmeric	MOL000449	Stigmasterol	PGR
Turmeric	MOL000449	Stigmasterol	NR3C2
Turmeric	MOL000449	Stigmasterol	NCOA2
Cicada slough	CT3	14-deoxy-11	RPS6KA5
Cicada slough	CT3	14-deoxy-11	SERPINA6
Cicada slough	CT3	14-deoxy-11	JAK2
White stiff silkworm	BJC12	beauverilide a	REN
White stiff silkworm	BJC12	beauverilide a	PSEN2
White stiff silkworm	BJC12	beauverilide a	OPRK1
...	...	...	...

TCM = traditional Chinese medicine.

### 3.2. Targets prediction for acne

A total of 3454 acne-related targets were retrieved in GeneCards, Comparative Toxicogenomics database, and Online Mendelian Inheritance In Man databases, and some genes are shown in Table 2.

**Table 2:** Acne-related genes.

Number	Gene symbol name	Number	Gene symbol name
1	CYP3A4	11	TNF
2	LHB	12	CCL2
3	F7	13	F2
4	CXCL8	14	ABCB1
5	VEGFA	15	SERPINE1
6	AR	16	ESR1
7	ABCB11	17	ACE2
8	PLAT	18	SERPINC1
9	FSHB	19	IL6
10	PROS1	...	...

### 3.3. Potential targets of PAD in the treatment of acne

**Figure 1.**
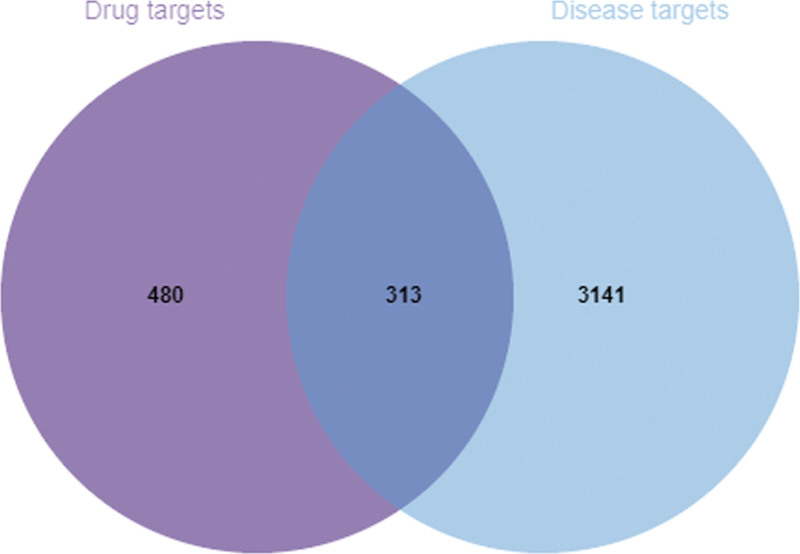
Venn diagram of the interactive targets of PAD and acne. PAD = powder ascending and descending.

The 793 ingredients-related target genes and 3454 acne-associated targets were imported into the Venn 2.1 software to draw the Venn diagram (Fig. 1). Among them, 313 were common targets of PAD and acne. This is the potential targets of PAD in the treatment of acne.

### 3.4. PPI network and core targets

**Figure 2.**
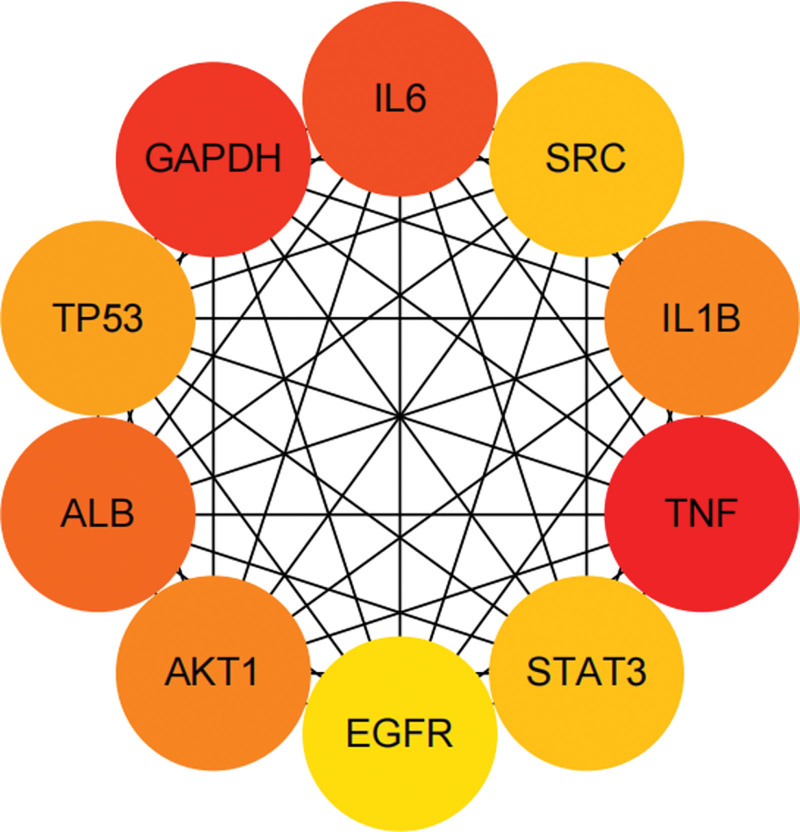
Network diagram in the treatment of acne with PAD. PAD = powder ascending and descending.

**Figure 3.**
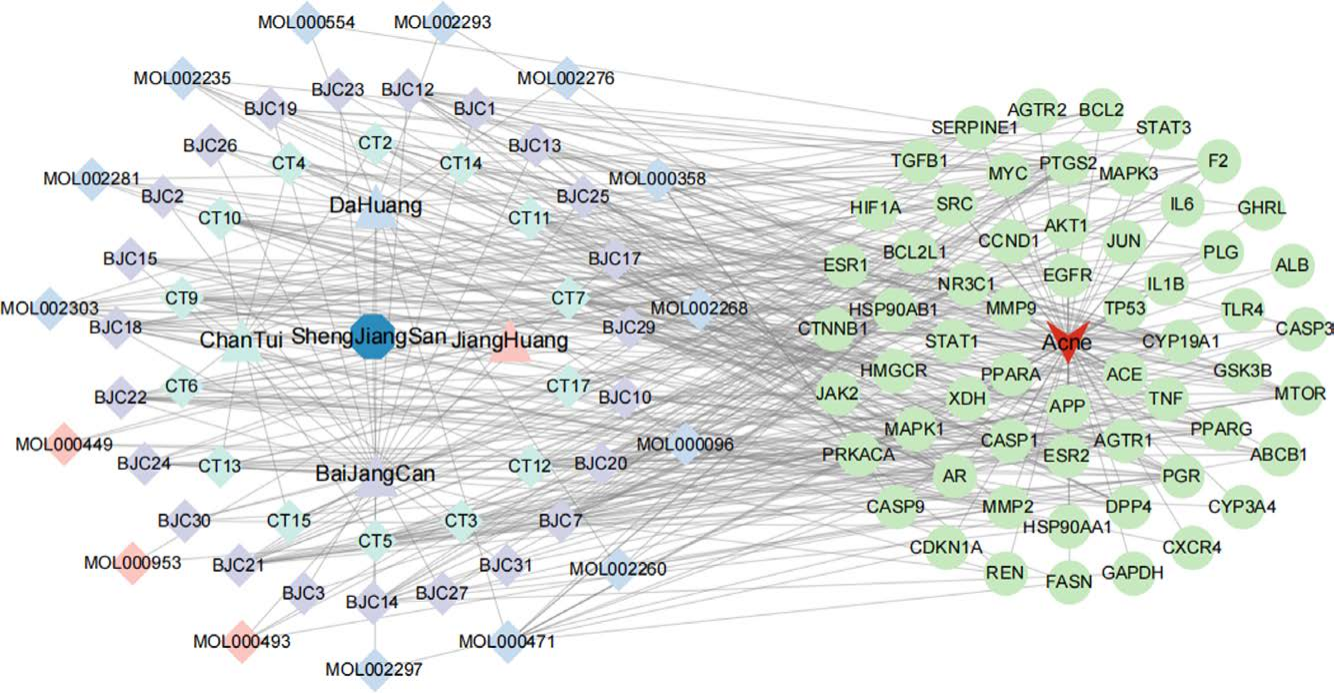
“Drug-component-target-disease” network diagram.

The above 313 potential targets were uploaded to the String PPI analysis platform, and the protein-protein relationships were saved in TSV format. Cytoscape software was imported to construct a PPI network, as shown in Figure 2. The cytoHubb plug-in in the software was used to carry out the network topology, and the degree value, intermediate center value and close relationship value of each node in the PPI network for the treatment of acne were calculated, and the top 30 targets were intersected according to the scoring values of each parameter. As a result, a total of 59 core targets for the treatment of acne were obtained, as shown in Table 3. On this basis, a “drug-component-target-disease” network for the treatment of acne was constructed, as shown in Figure 3. Through the analysis of CytoNCA plug-in, it was found that the most important active components in PAD were cyasterone (degree = 16), ecdysone (degree = 15), deoxyandrographiside (degree = 14), aurantiamid (degree = 14), etc. According to the results of PPI network analysis, tumor necrosis factor (TNF), glyceraldehyde-3-phosphate dehydrogenase (GAPDH) and interleukin 6 (IL6) may be the key targets of PAD in the treatment of acne.

**Table 3:** Network topology parameters of core targets of PAD against acne.

Number	Name	Degree	Betweenness Centrality	Closeness Centrality
1	TNF	175	0.041971012	0.691796009
2	GAPDH	173	0.061861193	0.688741722
3	IL6	172	0.039401597	0.685714286
4	ALB	161	0.045171615	0.670967742
5	AKT1	159	0.03296116	0.666666667
6	IL1B	159	0.03528227	0.666666667
7	TP53	143	0.022490595	0.641975309
8	STAT3	141	0.023058401	0.639344262
9	SRC	141	0.057512211	0.63803681
10	EGFR	133	0.018977824	0.63030303
...	...	...	...	...

PAD = powder ascending and descending, TNF = tumor necrosis factor.

### 3.5. Key targets gene enrichment analysis

**Figure 4.**
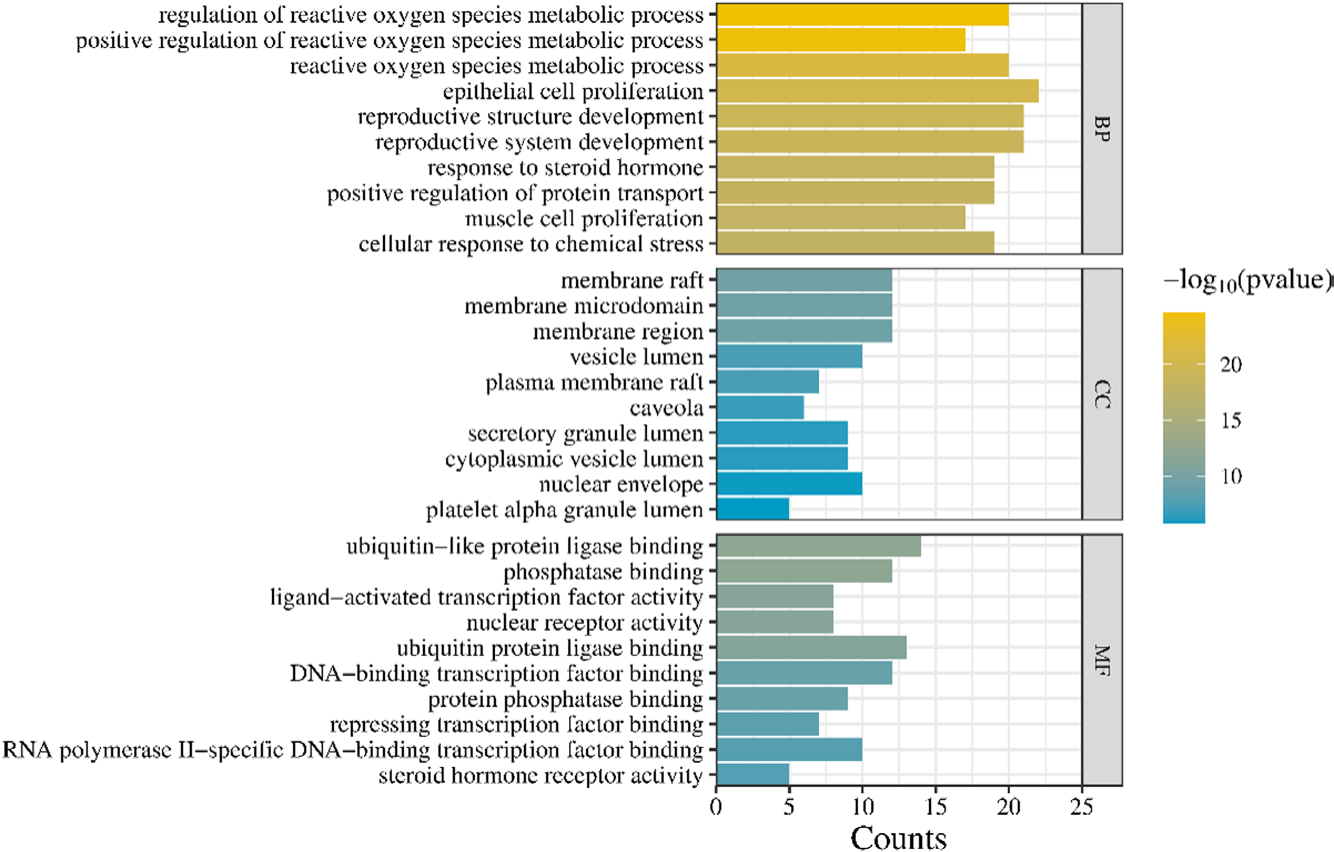
Bar graph of key targets GO functional enrichment analysis. GO = gene ontology.

PAD-acne intersection genes were analyzed by Metascape database, and the results with a *P* value < .05 were selected for statistics. The top 10 items with *P* values in Biological Process, Cellular Component, and Molecular Function were selected to draw bar charts, as shown in Figure 4. The Biological Process entries in the GO enrichment analysis of the core target genes of PAD treatment for acne mainly involved the regulation of reactive oxygen species metabolism, epithelial cell proliferation, reproductive structure development, reproductive system development, response to steroid hormones, positive regulation of protein transport, muscle cell proliferation, cell response to chemical stress, etc. Cellular Component-related items mainly include: membrane raft, membrane microdomain, membrane area, vesicular cavity, plasma membrane raft, membrane fovea, etc. Molecular Function-related items mainly include: ubiquitin-like protein ligase binding, phosphatase binding, nuclear receptor activity, ligand-activated transcription factor activity, ubiquitin protein ligase binding, DNA-binding transcription factor binding, protein phosphatase binding, inhibitory transcription factor binding, RNA polymerase II-specific DNA-binding transcription factor binding, steroid hormone receptor nuclear receptor activity, and so on.

**Figure 5.**
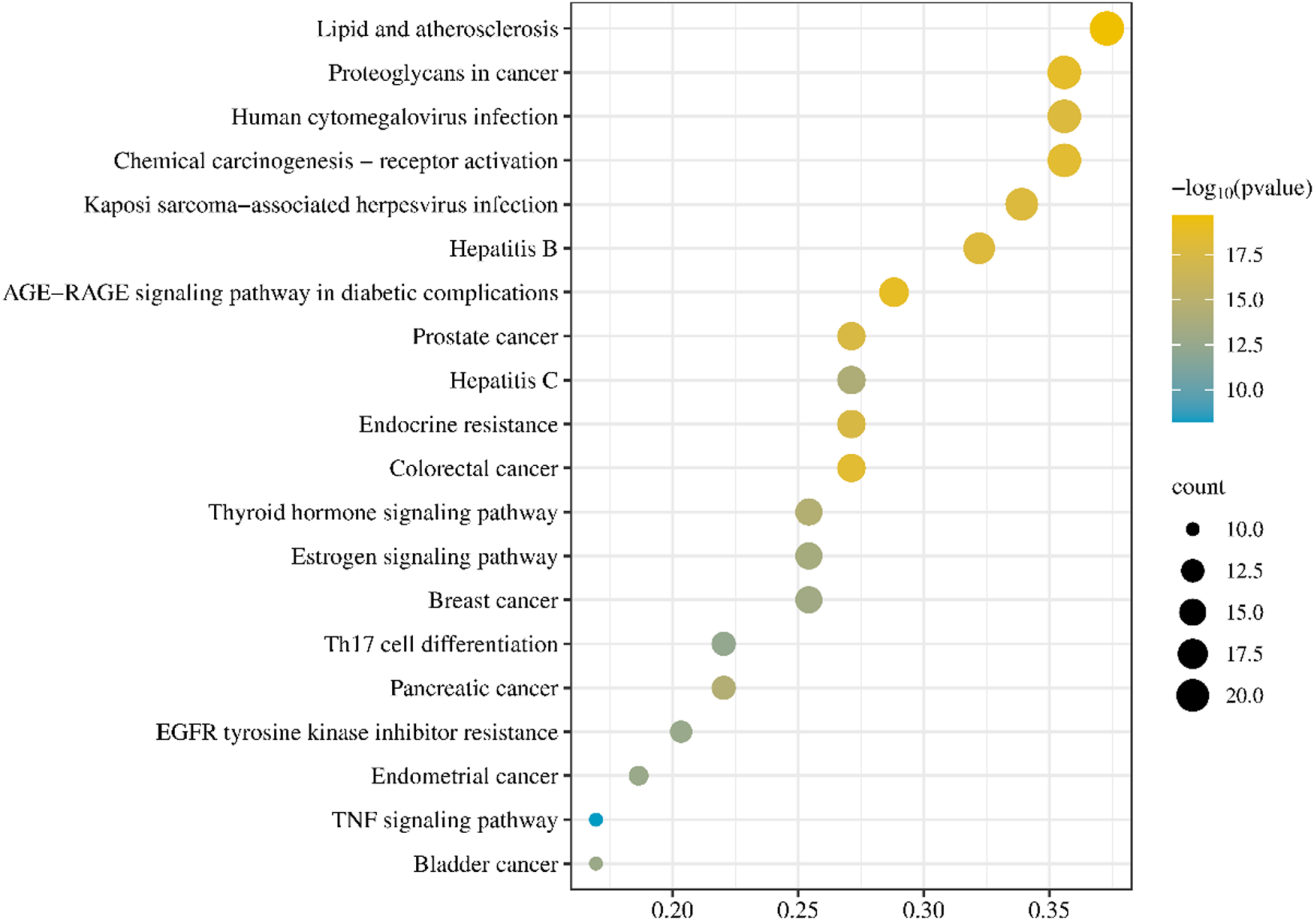
Bubble map of the core targets KEGG pathway enrichment analysis. KEGG = Kyoto Encyclopedia of Genes and Genomes.

**Figure 6.**
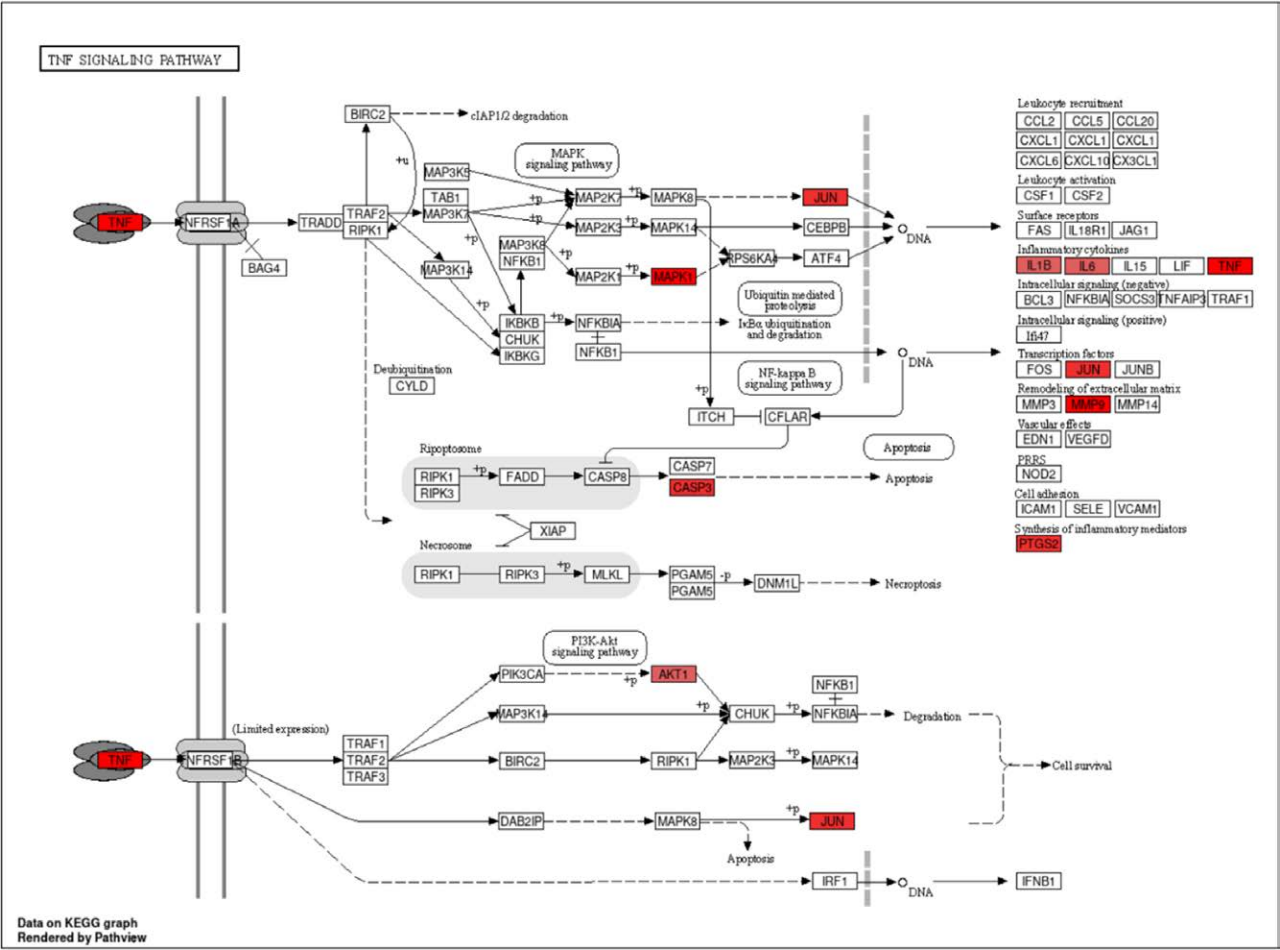
TNF signaling pathway map of potential target genes. TNF = tumor necrosis factor.

The results of KEGG pathway enrichment analysis of core target genes in PAD treatment of acne showed that lipids and atherosclerosis, AGE-RAGE signaling pathway, proteoglycans in cancer, chemocarcinogen-receptor activation, colorectal cancer, hepatitis B, human cytomegalovirus infection, Kaposi’s sarcoma-associated herpes virus infection, prostate cancer, endocrine resistance, thyroid hormone signaling pathway, pancreatic cancer, hepatitis C, estrogen signaling pathway, breast cancer, brace cancer, endometrial cancer, EGFR tyrosine kinase inhibitor resistance, Th17 cell differentiation, TNF and other signaling pathways contained high rates of enriched genes and large *P* values, as shown in Figure 5. In this study, the TNF signaling pathway will be further explored as a key pathway, as shown in Figure 6.

### 3.6. Molecular docking validation analysis of pharmaceutical ingredients and core targets

**Figure 7.**
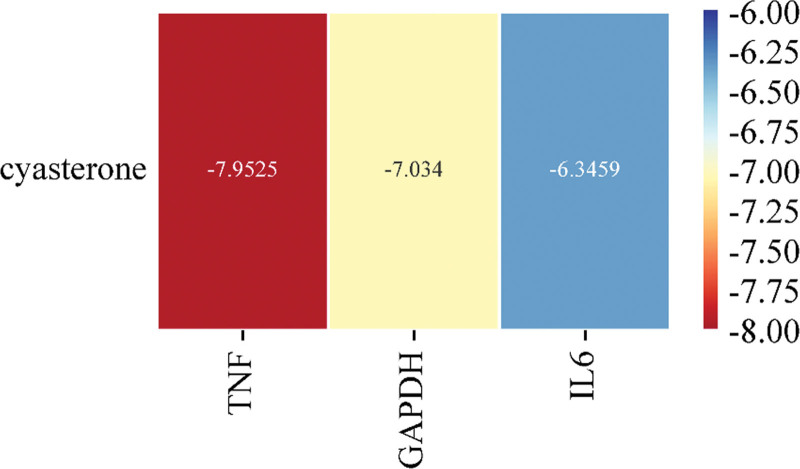
Heatmap of the binding capacity of key targets to cupacrylic acesterone.

**Figure 8.**
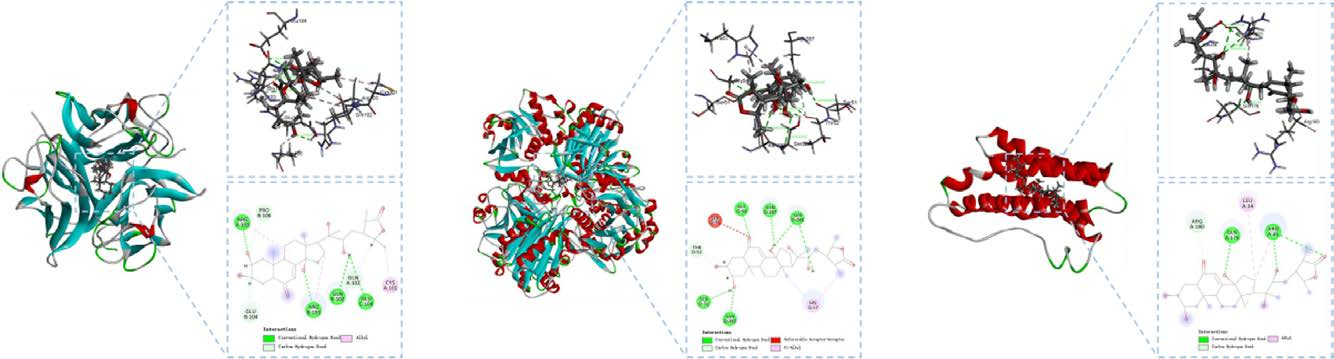
Molecular models of the binding of (L): TNF with cupacrylic acesterone; (M): GAPDH with cupacrylic acesterone; (R): IL-6 with cupacrylic acesterone. GAPDH = glyceraldehyde-3-phosphate dehydrogenase, IL-6 = Interleukin-6, TNF = tumor necrosis factor.

The top 3 core targets of PAD in the treatment of acne, TNF, GAPDH, and IL6 were selected for molecular docking with the main active ingredient cupacrylic carasterone, and the binding energy of each component to the protein was obtained, and the heat map was drawn as show in Figure 7. The results showed that all the affinities were < −5Kcal/mol, indicating that the receptors and ligands had strong binding activity and could bind spontaneously. The receptor proteins was molecularly docked to the ligands, and the results were shown in Figure 8. Among them, the binding force of cupacrylic acesterone to TNF was the lowest, which was −7.95254 kcal·mol^−1^ (1 kcal ≈ 4.186 kJ).

## 4. Discussion

Acne is a very common condition, especially among adolescents. While acne rarely leads directly to death, it can lead to the permanent formation of facial scars, which can have a great impact on the patient’s psychological and quality of life.^[8, 9]^ Various antibiotics have been widely used in the treatment of acne for many years because of their anti-inflammatory properties, which can effectively improve the clinical symptoms of acne patients, however, overuse of topical and/or systemic antibiotics can lead to the emergence of resistant strains.^[10]^ The PAD mentioned in this study has been clinically proven to be effective in controlling acne symptoms. The theoretical concept of network pharmacology is consistent with the multi-component and multi-target of TCM, and can be used as an important method for the analysis of drugs, components and disease targets. In this study, a total of 52 compounds were screened through TCMSP and HERB databases, including cupacrylic cyasterone (degree = 16), ecdysone (degree = 15), deoxyandrographiside (degree = 14), deoxyandrographolide (degree = 14), Aurantiamide (degree = 14) were identified as core active compounds. Among them, cupacrylic alasterone is the compound with the most targets, followed by ecdysone, so it is speculated that cupacrylic alasterone and ecdysone are the key compounds in PAD.

Cupacrylic cyasterone is a class of steroidal compounds with a structure similar to phytoestrogens β ecdyssterone, whose estrogen-like effects can reduce sebaceous gland secretion.^[11]^ Studies have shown that cupacrylic cyasterone inhibits the production of IL-1β-inducible nitric oxide synthase, cyclooxygenase-2, recombinant a disintegrin and metalloproteinase with thrombospondin 5, matrix metalloproteinase-3 (MMP-3), and matrix metalloproteinase-13 through nuclear factor-κB (NF-κB) and mitogen-activated protein kinase (MAPK) signaling pathways.^[12]^ As one of the important steroid hormones, ecdysone directs the spatial and temporal regulation of gene transcription through nuclear hormone receptors, preventing cell and tissue loss.^[13]^ Deoxyandrographins and deoxyandrographolides are natural inflammatory inhibitors and antioxidants^[14]^ that can inhibit the inflammatory response by inhibiting the activation of nuclear NF-κB and signal transducer and activator of transcription 3.^[15]^ Aurantiamide can inhibit the activation and expression of NOD-like receptor thermal protein domain associated protein 3 inflammasome, reduce central nervous system inflammation, and improve the level of inflammation in the body.^[16]^ In addition, Aurantiamide also effectively reduced the expression of MAPK2, MMP2 and prostaglandin-endoperoxide synthase 1 (PTGS1) in monosodium urate-stimulated THP-9 cells, and significantly inhibited the mRNA expression of caspase-2, spleen tyrosine kinase and PTGS1.^[17]^

In our study, a total of 313 active ingredients of PDA and their corresponding targets for the treatment of acne were obtained. Based on the PPI network analysis and topology algorithm in Cytoscape software, TNF, GAPDH, IL6, Albumin (ALB), and protein kinase 1 (AKT1) are considered to be the core target proteins for the treatment of acne. The elevated expression levels of TNF and IL6 as key inflammatory factors can accelerate the inflammatory response in tissues.^[18]^ TNF stimulates an intracellular signaling cascade to activate MAPK, which are highly active and involved in the production of pro-inflammatory mediators.^[19]^ TNF can also activate MMP for tissue remodeling.^[20]^ The cytokines of the IL-6 family are widely expressed long-chain quadruhelix bundle signal transduction proteins that bind to their corresponding cytokine-specific receptor and the ubiquitous signal transduction single-channel transmembrane receptor gp130 (CD130) and are activated,^[21]^ thereby regulating innate and adaptive immunity, inflammatory responses, early development, glucose and lipid metabolism, and more.^[22]^ In the nucleus, GAPDH is a core component of the transcript of genes involved in cell division. GAPDH binds to nucleic acids and is involved in mediating the export of RNA nuclei, thereby affecting mRNA stability and DNA damage repair.^[23]^ GAPDH is also a key mediator of cellular responses to oxidative stress and can directly promote apoptosis.^[24]^ ALB in serum is known as the “intravascular transporter,” which can be combined with various ions, hormone molecules and drug molecules to participate in a variety of pathophysiological processes, in addition to ALB also has the effect of stabilizing colloidal osmotic pressure, anti-inflammatory and antioxidative stress.^[25]^ A good immune status and effective control of inflammation can help prevent or reduce acne symptoms. AKT1 is a protein kinase belonging to the P13 kinase family that regulates metabolism, proliferation, survival, growth, and angiogenesis in normal and malignant cells.^[26]^ Activation of AKT1 is dependent on the PI3K pathway, which is a key component of many signaling pathways,^[27]^ and the signals downstream of PI3K mediate various growth factors, such as platelet-derived growth factor, epidermal growth factor, insulin-like growth factor-1, among which platelet-derived growth factor is released by a variety of cells in the early stage of injury, promotes fibroblast proliferation and collagen synthesis, and can accelerate wound healing.^[28]^

According to GO functional enrichment analysis, the treatment of acne involves a variety of molecular functions and biological processes. Biological processes mainly involve the regulation of reactive oxygen species metabolism, epithelial cell proliferation, reproductive system development, response to steroid hormones, and so on. These biological processes are known to regulate endocrine, antibacterial, and antioxidant processes, thus achieving the effect of treating acne. In this study, the KEGG pathway was enriched, and it was found that the target proteins of PAD in the treatment of acne were mainly enriched in the TNF, AGE-RAGE, HIF-1 signaling pathway.

The TNF signaling pathway is one of the important pathways that mediate inflammation and is mainly produced by monocytes and lymphocytes.^[29]^ TNF-α is considered to be the primary regulator of a variety of inflammatory and immune processes in human skin diseases, inducing specific chemokines to attract neutrophils and macrophages to the skin early in inflammation, in addition, TNF-α also affects many other cellular processes, including tissue repair, cytoskeletal changes, cell migration, keratinogenesis, and cell differentiation processes.^[30]^

The AGE-RAGE signaling pathway activates the expression of IL-6 and NF-κB signaling pathways and induces inflammatory responses while activating downstream pathways.^[14]^ The effects of the estrogen pathway on acne are multifaceted, including regulating sebum secretion, maintaining healthy stratum corneum, and anti-inflammatory effects.^[31]^ Estrogen can be synthesized locally in the skin, promote keratinocyte and fibroblast function, induce stromal deposition and promote anti-inflammatory (M2) cytokines, and conversely inhibit pro-inflammatory (M1) cytokines, thereby inhibiting inflammation and reducing wound protease levels.^[32]^ Similarly, estrogen has been shown to reduce the expression of pro-inflammatory cytokines and promote the transition of macrophages from M1 to M2.^[32]^

The molecular docking results showed that the key pharmacodynamic substances in PAD could be stably combined with the core target proteins TNF, GAPDH and IL6, indicating that PAD may act on TNF, GAPDH, IL6 and other targets, inhibit inflammatory responses, regulate cell proliferation and apoptosis, reduce the level of inflammatory factors, and regulate humoral and cellular immunity, so that it can be used for the treatment of acne.

## 5. Conclusion

Based on network pharmacology and molecular docking, this study studied the active ingredients, targets and pathways of PAD, clarified the role of the formula in the treatment of acne with multiple targets and pathways, and preliminarily revealed the molecular mechanism of PAD in the treatment of acne, which laid a foundation for subsequent in-depth research. Since TCM compound is a complex system with multiple components, and the occurrence of acne also involves many aspects, follow-up studies can be clinically and experimentally verified on the basis of the prediction of the above targets and signaling pathways, so as to make its application more instructive.

Overall, TCM treatment strategies are quite similar to Western medical treatment concepts, with a focus on anti-inflammatory, antibacterial, anti-hyperkeratosis and reducing sebum production.

## Author contributions

**Conceptualization:** Ruyi Zheng.

**Funding acquisition:** Zukang Qiao.

**Investigation:** Ruyi Zheng.

**Methodology:** Jiani Wu.

**Software:** QianJun Yan, Jiani Wu.

**Supervision:** Zukang Qiao.

**Validation:** Zukang Qiao.

**Visualization:** Yangzi Jin.

**Writing – original draft:** QianJun Yan.

**Writing – review & editing:** Fang Zhang.

## References

[1] Williams HC Dellavalle RP Garner S. Acne vulgaris. Lancet. 2012;379:361–72. 21880356 10.1016/S0140-6736(11)60321-8

[2] Xu H Li H. Acne, the skin microbiome, and antibiotic treatment. Am J Clin Dermatol. 2019;20:335–44. 30632097 10.1007/s40257-018-00417-3 PMC6534434

[3] Habeshian KA Cohen BA. Current issues in the treatment of acne vulgaris. Pediatrics. 2020;145:S225–30. 32358215 10.1542/peds.2019-2056L

[4] Eichenfield DZ Sprague J Eichenfield LF. Management of acne vulgaris: a review. JAMA. 2021;326:2055–67. 34812859 10.1001/jama.2021.17633

[5] Chun YC Ghuang YX Yan YS. Acupuncture: a therapeutic approach against acne. J Cosmet Dermatol. 2021;20:3829–38. 34599626 10.1111/jocd.14487

[6] Chen HY Lin YH Chen YC. Identifying Chinese herbal medicine network for treating acne: Implications from a nationwide database. J Ethnopharmacol. 2016;179:1–8. 26721214 10.1016/j.jep.2015.12.032

[7] Zhi CW Jing C Fang Z. Traditional Chinese medicine has great potential as candidate drugs for lung cancer: a review. J Ethnopharmacol. 2023;10:0378–41. 10.1016/j.jep.2022.115748 36162545

[8] Drake L Reyes HS Barbieri JS. New developments in topical acne therapy. Am J Clin Dermatol. 2022;23:125–36. 35041198 10.1007/s40257-021-00666-9

[9] Dessinioti C Katsambas A. Antibiotics and antimicrobial resistance in acne: epidemiological trends and clinical practice considerations. Yale J Biol Med. 2022;95:429–43. 36568833 PMC9765333

[10] Yi J Luo Y Li B Zhang G. Phytoecdysteroids and glycoceramides from Eriophyton wallchii. Steroids. 2004;69:809–15. 15582536 10.1016/j.steroids.2004.08.002

[11] Teng L Shen Y Qu Y. Cyasterone inhibits IL-1β-mediated apoptosis and inflammation via the NF-κB and MAPK signaling pathways in rat chondrocytes and ameliorates osteoarthritis in vivo. Chin J Nat Med. 2023;21:99–112. 36871986 10.1016/S1875-5364(23)60388-7

[12] Xu T Jiang X Donna D. Ecdysone controlled cell and tissue deletion. Cell Death Differ. 2020;27:1–14. 31745213 10.1038/s41418-019-0456-9 PMC7205961

[13] Gu L Lu L Li Q. A network-based analysis of key pharmacological pathways of Andrographis paniculata acting on Alzheimer’s disease and experimental validation. J Ethnopharmacol. 2020;251:112488. 31866509 10.1016/j.jep.2019.112488

[14] Janam P Prasad M Vinod D. An ex vivo evaluation of the efficacy of andrographolide in modulating differential expression of transcription factors and target genes in periodontal cells and its potential role in treating periodontal diseases. J Ethnopharmacol. 2017;196:160–67. 27993634 10.1016/j.jep.2016.12.029

[15] Shen H Pei H Zhai L Guan Q Wang G. Aurantiamide suppresses the activation of NLRP3 inflammasome to improve the cognitive function and central inflammation in mice with Alzheimer’s disease. CNS Neurosci Ther. 2023;29:1075–85. 36627760 10.1111/cns.14082 PMC10018077

[16] Ye X Wu J Zhang D. How aconiti radix cocta can treat gouty arthritis based on systematic pharmacology and UPLC-QTOF-MS/MS. Front Pharmacol. 2021;12:618844. 33995019 10.3389/fphar.2021.618844 PMC8121251

[17] Ng A Tam WW Zhang MW. IL-1β, IL-6, TNF- α and CRP in elderly patients with depression or alzheimer’s disease: systematic review and meta-analysis. Sci Rep. 2018;8:11–22. 30104698 10.1038/s41598-018-30487-6 PMC6089986

[18] Heng AHS Say YH Sio YY Ng YT Chew FT. Gene variants associated with acne vulgaris presentation and severity: a systematic review and meta-analysis. BMC Med Genomics. 2021;14:11–27. 33849530 10.1186/s12920-021-00953-8 PMC8045239

[19] Zelová H Hošek J. TNF-α signalling and inflammation: interactions between old acquaintances. Inflamm Res. 2013;62:641–51. 23685857 10.1007/s00011-013-0633-0

[20] Murakaki M Kamimura D Hirano T. Pleiotropy and specificity: insights from the interleukin 6 family of cytokines. Immunity. 2019;50:812–31. 30995501 10.1016/j.immuni.2019.03.027

[21] Dawson RE Jenkins BJ Saad MI. IL-6 family cytokines in respiratory health and disease. Cytokine. 2021;143:155520. 33875334 10.1016/j.cyto.2021.155520

[22] Zheng L Zheng HL Liu XG. GAPDH as a housekeeping gene: analysis of GAPDH mRNA expression in a panel of 72 human tissues. Chin J Biochem Mol Biol. 2018;4:385–89.

[23] Zhang YL Zhang F Hong CQ. Critical protein GAPDH and its regulatory mechanisms in cancer cells. Cancer Biol Med. 2015;12:11–23. 10.7497/j.issn.2095-3941.2014.0019 PMC4383849 25859407

[24] Arques S Ambrosi P. Human serum albumin in the clinical syndrome of heart failure. J Card Fail. 2011;17:451–58. 21624732 10.1016/j.cardfail.2011.02.010

[25] Shi L Yang YB Huang YJ. Effects of Jianpi Huashi Formula on PI3K/Akt pathway in skeletal muscle of obese type 2 diabetic insulin resistance rats with syndrome of spleen deficiency and phlegm-dampness. Chin J Tradit Chin Med. 2018;33:530–34.

[26] Revathidevi S Munirajan AK. Akt in cancer: mediator and more. Semin Cancer Biol. 2019;59:80–91. 31173856 10.1016/j.semcancer.2019.06.002

[27] Yamane K Ihn H Asano Y Jinnin M Tamaki K. Antagonistic effects of TNF-alpha on TGF-beta signaling through down-regulation of TGF-beta receptor type II in human dermal fibroblasts. J Immunol. 2003;171:3855–62. 14500687 10.4049/jimmunol.171.7.3855

[28] Sethi G Sung B Aggarwal BB. TNF: a master switch for inflammation to cancer. Front Biosci. 2008;13:5094–107. 18508572 10.2741/3066

[29] Choi JJ Park MY Lee HY. TNF-α increases lipogenesis via JNK and PI3K/Akt pathways in SZ95 human sebocytes. J Dermatol Sci. 2012;25:179–88. 10.1016/j.jdermsci.2011.11.005 22305016

[30] Wilkinson HN Hardman MJ. The role of estrogen in cutaneous ageing and repair. Maturitas. 2017;103:60–4. 28778334 10.1016/j.maturitas.2017.06.026

[31] Labrie F. All sex steroids are made intracellularly in peripheral tissues by the mechanisms of intracrinology after menopause. J Steroid Biochem Mol Biol. 2015;145:133–8. 24923731 10.1016/j.jsbmb.2014.06.001

[32] Routley CE Ashcroft GS. Effect of estrogen and progesterone on macrophage activation during wound healing. Wound Repair Regen. 2009;17:42–50. 19152650 10.1111/j.1524-475X.2008.00440.x

